# Gender and race in neurotrauma: part 1-identifying inequalities in leadership, academics, and clinical trial management

**DOI:** 10.3389/fneur.2024.1383713

**Published:** 2024-10-31

**Authors:** Isabella F. Churchill, Téa Sue, Ann M. Parr, Eve C. Tsai

**Affiliations:** ^1^Faculty of Medicine, University of Ottawa, Ottawa, ON, Canada; ^2^Department of Neurosurgery, University of Minnesota, Minneapolis, MN, United States; ^3^Division of Surgery, Department of Surgery, Faculty of Medicine, University of Ottawa, Ottawa, ON, Canada; ^4^Neuroscience Program, Ottawa Hospital Research Institute, Ottawa, ON, Canada

**Keywords:** clinical trial, gender, diversity, leadership, academics

## Abstract

Gender and racial equality, or the lack thereof, is a constantly recurring theme in neurosurgery and under-reported in neurotrauma literature. This perspective piece addresses the underrepresentation and challenges faced by women and racial minorities in neurosurgery, and within the workforce of neurotrauma, specifically. The literature demonstrates that there is still a scarcity of females and racial minorities in neurosurgery leadership positions and that females are less likely to receive invited papers. The persistent challenges in navigating gender and racial dynamics in neurosurgery/neurotrauma underscore the need for progress in advancing intersectionality within the field, emphasizing the importance of addressing inequalities. Several strategies to improve gender and racial diversity in neurotrauma workforce, leadership and academics are presented.

## Introduction

In 2008, the Board of the Directors of the American Association of Neurological Surgeons (AANS) collaborated with Women in Neurosurgery (WINS) to compose a white paper to promote the recruitment and retention of women in neurosurgery, given their historical status of being deemed “less than a minority” (1, 2). Women entering neurosurgical residency programs faced significant and disproportionate obstacles in their path to becoming surgical experts (2). Beyond neurosurgery, the surgical field was previously known to be dominated by Caucasian males, which is beginning to shift as women now make up most of medical school class graduates (3–8). Recent data suggests a stark increase in the number of women neurosurgical residents. For a while, such disparity was attributed to numerous factors including the perceived department culture, lack of concordant mentors and the “pipeline effect,” where poor female representation in the upper levels of academic surgery was explained by the small candidate pool (3, 4, 9, 10). However, persistence of the gap despite females beginning to occupy a higher proportion of the surgical workforce, indicates other contributors to this ongoing issue (3, 6, 8, 9). Lack of diversity in the workforce and among those involved in other academic leadership and research endeavors have both direct and indirect impacts on the quality of patient care. It may lead to the direct oversight of the unique needs and challenges faced by certain subsets of the population and indirectly, the narrow range of perspectives discussed may hinder innovation and development of inclusive policies. As the field of neurosurgery makes efforts to increase the numbers of female surgeons, there is an evident need to concurrently adapt the surrounding culture to the new wave of trainees while attending to the inherent biases ingrained into the fabric of the field (2). In this paper, we present an overview of the current state and progression of gender and racial diversity in neurosurgery, with a specific focus on the neurotrauma workforce. Additionally, we offer a compilation of recommendations aimed to both inspire interested individuals to pursue their passion in the evolving environment.

## Neurosurgery leadership underrepresentation

The underrepresentation of women and racial minorities in neurosurgical leadership is a global concern that demands attention. Notably, there is a lack of studies specifically addressing gender or racial diversity within neurotrauma leadership, highlighting a significant gap in research. Female neurosurgeons face challenges in obtaining leadership positions within organizational neurosurgery globally, both within surgical societies and among institutional academic ranks. Several studies have investigated the underrepresentation of these groups and placed an emphasis on the importance of acknowledging and addressing this disparity, encouraging neurosurgical societies worldwide to take proactive steps to mitigate these problems. A cross-sectional study examining the representation of women on neurosurgical editorial boards revealed that only approximately 9% of positions are held by women, a percentage comparable to the number of practicing female academic neurosurgeons (11). This finding suggests that biases in the selection of editorial board members may not be readily identifiable, but efforts to recruit and retain women in neurosurgery are crucial to rectify existing discrepancies.

It has also been well documented that there is a scarcity of women in leadership positions within academic neurosurgery. Notably, in 2011 Dr. Karin Muraszko, was the first and only female chair of a neurosurgical department (3) and over a decade later, there are currently only three female department chairs of a neurosurgery, Drs. Ellen Air (Detroit), Aviva Abosch (Nebraska) and Linda Liau (UCLA). Furthermore, Dr. Shelley Timmons (Illinois) is the only former female chair who has also been a neurotrauma specialist. The data reinforces that women constitute a minority in neurosurgical leadership, accounting for less than 15% of total opportunity spots at major neurosurgical conferences over a 5-year period (12). Additionally, there had never been a female president for any major neurosurgical society until 2019. The American Association of Neurological Surgery was notably male dominated, highlighting persistent gender disparities in leadership roles. Additionally, female neurosurgeons make up just 6.3% (1,024/16294) of the American Association of Neurological Surgeons (AANS) members (13). However, global efforts by committees such as the Women in Neurosurgery Committee of the World Federation of Neurosurgical Societies and the Task Force on Diversity established by the European Association of Neurosurgical Societies are actively working toward addressing gender diversity in neurosurgery (14, 15).

## Discordance in neurosurgery authorship

The underrepresentation of women and minority authors in neurosurgery publications remains a persistent challenge, even as the overall number of underrepresented neurosurgeons have increased. While studies have investigated various aspects of gender diversity within neurosurgery such as authorship patterns and research productivity, there is a notable gap in research focusing on the racial backgrounds of neurosurgeon authors. The literature lacks comprehensive data specifically analyzing racial/ethnic minority authorship rates in neurosurgery and neurotrauma publications as most studies focus solely on gender disparities in authorship. Several studies have reported a significant increase in female authorship in high-impact neurosurgical research publications, particularly in the United States and Canada (16). Despite this progress, disparities persist as female authorship rates remain low at 13.4% (*n* = 570) for first authors and 6.8% (*n* = 240) for last authors, suggesting potential biases in collaboration patterns (17). Interestingly, no significant difference is found between single-blind and double-blind peer review processes (17). There is also evidence that female first authors tend to collaborate with female senior authors (18). Data from 2015 to 2019 indicates a modest rise in the number of female neurosurgeons. However, the representation of women as authors in neurosurgical and spine journals remains strikingly low, with only 8.3 and 5.8% serving as first and last authors, respectively (13). In spine specific research, the representation and longevity of female physician-investigators in spine-related research journals from 1978 to 2016 was investigated and showed doubling of female representation, especially as first authors (19). However, this growth plateaus after the year 2000, and female physician-investigators are less likely to continue participating in spine-related research, publishing fewer articles compared to their male counterparts.

## Discussion

This compilation of studies serves as a pivotal contribution to the neurotrauma research landscape, directing its focus to diversity. The significance of this endeavor lies in its targeted effort to fill a substantial knowledge gap that has persisted within the field. By concentrating specifically on women and minorities, these studies illuminate aspects of neurotrauma that have historically received inadequate attention, thereby enriching the overall discourse on diversity in neurotrauma. The application of a gender and a racial lens is particularly noteworthy, as it goes beyond mere recognition of differences and actively seeks to comprehend the influence of social structures on a spectrum of outcomes. Crucially, these articles bring to the forefront the challenges associated with grappling with the intricacies of gender and racial dynamics within the broader domain of neurotrauma research. In essence, these studies represent the need for a crucial step forward in advancing intersectionality in this field.

The critical relationship between leadership diversity and patient access to various opportunities, including clinical trial participation, cannot be overlooked. Unrepresentative leadership has known adverse consequences, including compromised scientific integrity where results cannot be generalized beyond the stated study population, negatively implicating patients who will not benefit from such research investments (20). However, a positive relationship between enhanced leadership diversity and trial enrollment was demonstrated by Chhaya et al. (21). In this study, trials with female first and senior authors had a significantly higher proportion of female clinical trial participants, suggesting a promising trend toward increased female trial enrollment over time as support for female and minority scientists to take on these roles grows (21).

With respect to diversity in research, it is important to analyze representation among researchers. Although women have begun to occupy more academic surgical leadership positions, they remain a minority. Only 19% of academic surgery faculty are female (5) and women were reported to make up only 8% of professors, 13% of associate professors and 26% of assistant professors of surgery (5, 22). Unfortunately, the many factors considered when determining academic promotion including research productivity and occupation of academic leadership positions, are known to be influenced by gender (5, 22). Prior studies have identified women to hold fewer positions on journal editorial boards, to have fewer publications, and to advance in academic rank slower when compared to their male counterparts (3, 5, 6, 23). This is further impacted by a prior concern for a self-perpetuating bias where grant funding is preferentially awarded to experienced surgeons, many of whom are male (24). In a study by Krebs et al. (5), women were found to hold 26.4% of the National Institute of Health (NIH) R01 grants, both a minority and an overrepresentation of the proportion of women in academic surgery. Interestingly, female primary investigators were twice as likely to be first-time recipients and to have obtained the grant in the last 5 years, suggesting a more recent shift toward improved female representation in R01 funding (5). Furthermore, these individuals were more likely to come from departments with a high proportion of female chairs and faculty, potentially pointing to a supportive environment where success in leadership and research co-exist (5).

## Roadmap to closing the gender and racial gap in neurotrauma workforce

Despite women’s increased participation in traditionally male-dominated fields, the term “glass ceiling” remains a pervasive metaphor for the persistent underrepresentation of women in leadership roles in the absence of clear obstacles preventing their advancement (25, 26). The term “sticky floor” was further applied secondary to the realization that women were given fewer resources at the beginning of their career to support their ascent up the institutional ladder (25, 26). The presence of a glass ceiling and sticky floor in surgical specialties extends in part from the historical roles men and women have previously occupied in society, with little acknowledgement of the intellect and skill such individuals currently have to offer (26). To compound the inherent difficulty posed by traditional gender roles, women often face additional obstacles due to societal expectations of their role in managing household responsibilities (25, 26). Such competing pressures and the clinical and training demands associated with surgical specialties disproportionately affect women and as a result, females are more likely to change the focus of their practice to accommodate familial responsibilities and are twice as likely to leave academia (27–30). Furthermore, the pipeline effect, characterized by the ongoing attrition of women and minorities at each career stage, has been proposed as an additional factor contributing to the diversity challenges in the field, with minority females being particularly affected (31). Among general surgery residents, increased incidences of racial discrimination have been linked to feelings of burnout and eventual attrition from the field, with Black residents, particularly Black females, facing the highest prevalence of discrimination (32–34). While such data is currently unavailable in the field of Neurosurgery, female neurosurgical residents are known to leave the specialty at a greater rate than their male counterparts (34, 35). It is therefore critical to acknowledge that efforts to address racial or gender biases in isolation may not be sufficient to attend to the unique barriers faced at the intersection of the two.

Mentors play a crucial role in guiding individuals toward personal and professional growth, and this role is not limited to the fields of academic surgery or medicine. Previous research has identified both the beneficial role demographically-concordant mentors play in career development and navigation of the health care system, and the role lack of mentorship plays as a deterrent to entering certain surgical fields (36–43). In agreement with the previous finding surrounding NIH funding, most female students in a study by Neumayer et al. demonstrated that those who pursued a surgical career graduated from medical schools with a higher proportion of female surgical faculty, suggesting the beneficial role of visible female role models and mentors (44). Therefore, the development and implementation of mentorship programs aimed at providing accessible and compatible opportunities are important to encourage female and minority students to both pursue their surgical interests and aspire for academic positions if so desired (4, 27, 37, 45, 46). Although a mentor who is demographically concordant can provide strategic advice for areas outside of medicine, including strategies for maintaining a healthy work-life balance, it is important to recognize the greater importance of a mentor’s desire to teach over similar demographics (5, 36). Therefore, further teaching on how to seek a mentor and how to be an effective mentor may improve both the quantity and quality of these relationships (36).

Although improvements to recruitment are critical to advancing the diversity of future generations of surgeons, integrating supports that promote surgeon retention and career-satisfaction are equally as important in maintaining a diverse workforce (46, 47). Sexism in the workplace has multiple manifestations from outright acts of harassment to more covert measures including omission of females in certain interactions, referral biases, and the inclination to question a women’s commitment to their career based on their dedication to their family (26, 47). Additionally, it is crucial to acknowledge the evident but often unspoken impact of structural racism, whereby policies, practices, and institutional norms perpetuate racial disparities, irrespective of individual actions (48, 49). Efforts to address acts of gender and racial discrimination cannot be carried out in silos as such acts are significantly impacted by the institutional environment, culture, and other social forces (39). However, simultaneously addressing the lack of parity in surgical leadership and mentorship accessibility may serve as a start to bring greater awareness to such issues (39). Providing and normalizing the use of protected personal time for familial responsibilities in addition to workshops that sensitize staff to common gender and racially-derived issues can help reduce implicit bias and foster a more inclusive environment that attracts and retains female and minority surgeons (26, 29, 46). Furthermore, establishing a diverse leadership group that encourages the reporting of offensive or discriminatory behavior may reduce the role of discrimination in hindering female progression in the field of academic surgery (26, 29).

Although a lot of work is required to approach parity in representation among faculty, within leadership and within research, it is important to acknowledge the steps that have been taken. Worldwide, surgical societies have implemented strategies to enhance organizational culture and consequentially improve both the recruitment and retention of female surgeons (22). Furthermore, organizations such as the Gender Equity Initiative in Global Surgery hope to address gender disparities in surgical specialties in low- and middle-income countries by 2030, with approaches from a variety of avenues (22). To build upon existing steps, Table 1 outlines our recommendations toward improving diversity among neurosurgeons, which have downstream effects on the associated culture, academic achievements, and patient care.

**Table 1:** Recommendations on how to improve diversity in neurotrauma workforce.

Workforce diversity	
Recommendations	Supporting evidence
Integrate leadership training into medical education	Integrating leadership training into core medical education would serve to empower females with the skills and confidence required to pursue academic leadership positions, ultimately leading to a greater proportion of strong, female leaders. Prior research suggests that enhanced female departmental leadership may contribute to a supportive environment conducive to applying for and receiving substantial research grants (5).
Improve demographically concordant mentorship opportunities	The evident lack of female neurosurgeons may foster feelings of loneliness and isolation in younger surgical trainees who do not have a demographically concordant role model to see themselves in or debrief unique experiences with (3). However, effective mentoring relationships do exist without demographic concordance, therefore, investing in robust mentorship programs that outline and address specific barriers the mentee may face as they enter the neurosurgical field would also be beneficial.
Adapt to the unique needs of female and minority surgeons	Establishing a flexible environment that supports the various roles and responsibilities of the residents and staff to both improve neurosurgeon recruitment and retention (2, 22) for individuals otherwise swayed by such factors.
Adjusting expectations of neurosurgical trainees and staff	Adjusting expectations and adapting to the growing priority on work-life balance may help attract young, competent and passionate trainees to a field historically perceived to inherently oppose such beliefs. With ongoing medical and technological advances, flexibility in educational and practice approaches may allow for better support for the improved work/life balance desired by some within the generations of neurosurgeons to come (3).
Identify and address biases inherent to the environment	Identify and attend to discriminatory practices within the multiple levels of neurosurgical recruitment, any of which may deter females and minorities from pursuing their desires to enter the field. Addressing such factors may aid in fostering a safer and more equitable review process that mitigates the strength of the well-ingrained biases and stereotypes associated with the field (22, 47, 50).

The surgical field has been predominantly composed of Caucasian males, with Neurosurgery being no exception (3). Improved diversity in the workforce has parallelled both the changing demographic of medical school class graduates and implementation of targeted efforts. Which is critical given the direct and indirect impacts lack of diversity has on patient care (2–8). As highlighted in this paper, female neurosurgeons hold fewer organizational leadership positions and compose a minority of first and last authorship positions in comparison to their male counterparts (11, 19). The articles discussed in this review explored how institutional structures and embedded norms influence gender and racial dynamics within neurotrauma and emphasize the significant gaps that remain despite an insurgence of aligned research. Although we are beginning to see a rise in the proportions of female neurosurgeons who occupy leadership or lead/senior author roles, several obstacles including societal expectations, the embedded culture, and lack of demographically concordant mentors, still exist. Efforts to address gender and racial discrimination are growing, however, solitary efforts may not be sufficient to address the unique barriers faced by individuals at the intersection of gender and racial minority status. To effectively enhance diversity in the neurotrauma workforce, individuals are encouraged to employ various strategies and guidelines (Table 1) to foster inclusivity and equitable representation.

## Data Availability

The original contributions presented in the study are included in the article/supplementary material, further inquiries can be directed to the corresponding author.
